# A Survey on Analog-to-Digital Converter Integrated Circuits for Miniaturized High Resolution Ultrasonic Imaging System

**DOI:** 10.3390/mi13010114

**Published:** 2022-01-11

**Authors:** Dongdong Chen, Xinhui Cui, Qidong Zhang, Di Li, Wenyang Cheng, Chunlong Fei, Yintang Yang

**Affiliations:** School of Microelectronics, Xidian University, Xi’an 710071, China; ddchen@xidian.edu.cn (D.C.); xhcuia@163.com (X.C.); qdzhang@xidian.edu.cn (Q.Z.); clfei@xidian.edu.cn (C.F.); ytyang@xidian.edu.cn (Y.Y.)

**Keywords:** analog-to-digital converter, ultrasonic imaging, miniaturized system, integrated circuit

## Abstract

As traditional ultrasonic imaging systems (UIS) are expensive, bulky, and power-consuming, miniaturized and portable UIS have been developed and widely utilized in the biomedical field. The performance of integrated circuits (ICs) in portable UIS obviously affects the effectiveness and quality of ultrasonic imaging. In the ICs for UIS, the analog-to-digital converter (ADC) is used to complete the conversion of the analog echo signal received by the analog front end into digital for further processing by a digital signal processing (DSP) or microcontroller unit (MCU). The accuracy and speed of the ADC determine the precision and efficiency of UIS. Therefore, it is necessary to systematically review and summarize the characteristics of different types of ADCs for UIS, which can provide valuable guidance to design and fabricate high-performance ADC for miniaturized high resolution UIS. In this paper, the architecture and performance of ADC for UIS, including successive approximation register (SAR) ADC, sigma-delta (Σ-∆) ADC, pipelined ADC, and hybrid ADC, have been systematically introduced. In addition, comparisons and discussions of different types of ADCs are presented. Finally, this paper is summarized, and presents the challenges and prospects of ADC ICs for miniaturized high resolution UIS.

## 1. Introduction

Ultrasonic imaging is one of the most widely used imaging methods in nondestructive testing and medical diagnosis due to its advantages of safety, simplicity, cheapness, penetrability, and non-ionization [1,2,3,4,5,6]. Due to the demand of high-resolution medical imaging equipment, ultrasonic imaging systems (UIS) have been rapidly developed and applied in many fields [7,8,9,10,11,12,13]. Generally, traditional UIS are expensive, large in size, heavy in mass, and have high power consumption. The miniaturized and portable UIS have been gradually developed and used in a variety of medical conditions such as ambulances, emergency rooms, and remote areas where large-scale ultrasound imaging equipment is not convenient [14,15,16,17,18]. However, higher resolution imaging is required for the future advanced ultrasonic Doppler imaging, 3D imaging, elastography, etc. The requirement for miniaturized high resolution UIS is urgent, and it has great significance to investigate high-resolution UIS [19,20,21,22].

**Figure 1:**
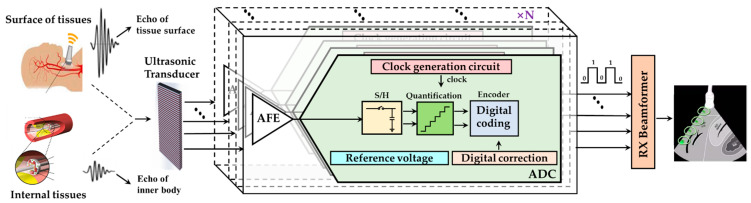
Schematic diagram of ultrasonic imaging receiver chain.

The integrated circuits (ICs) and external devices responsible for signal reception and data conversion have great influence on the comprehensive performance of the UIS. Without compromising imaging quality, the IC with small area, low power consumption, and high precision has become the mainstream trend [23,24,25,26,27]. A typical UIS receiving chain, as shown in Figure 1, includes analog front-end (AFE), which preprocesses the analog echo signal, and the analog-to-digital converter (ADC), which digitalizes the echo signal for further processing by the digital signal processing (DSP) or microcontroller unit (MCU) [28,29,30,31,32,33,34]. The ADC, which realizes the digitalization of the analog echo signals, plays a very important role in the receiving link. Its performance indexes, such as conversion accuracy, speed, linearity, etc., directly affect the quality of UIS. Therefore, according to the urgent requirements of miniaturized high resolution UIS, how to design and fabricate high-performance ADC ICs is very important.

In order to achieve the higher resolution and smaller volume for miniaturized UIS, ADC ICs integrated into the portable imaging terminals or even probes are confronted with high performance requirements:

(1) ADC is the main power consumption module in the UIS receiving chain. Power consumption exponentially increases with the channel number, and low power consumption becomes one of the main optimization directions of ADC.
(2) In remote districts, only miniaturized or portable medical equipment is available due to uneven medical development. High integration and small size of receiver link (including ADC) chips are urgently needed for advanced portable or handy UIS devices.
(3) Advanced UIS typically require high resolution and high-speed A/D data converters to improve ultrasonic imaging quality. Tradeoffs among sampling rate, signal to noise ratio (SNR), occupied area, and power consumption must be carefully considered.

**Figure 2:**
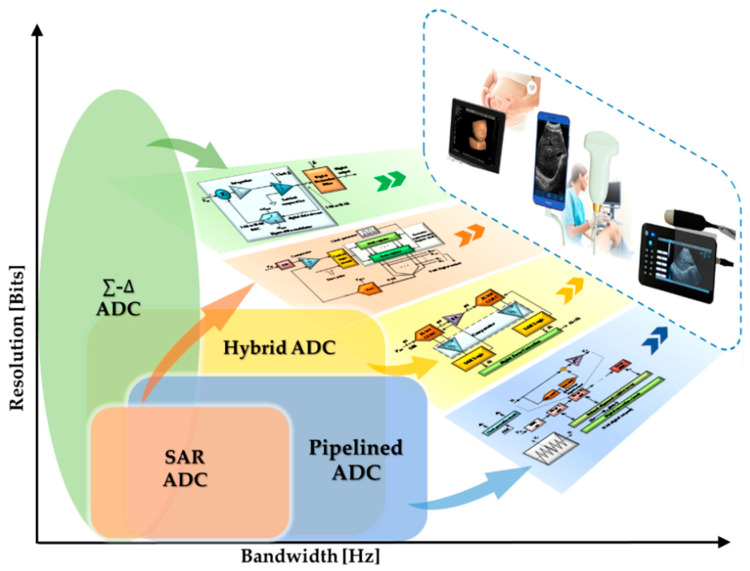
Structures of ADC in miniaturized high resolution UIS.

In this paper, the recent development and prospect of ADC ICs for miniaturized high resolution UIS are reviewed to provide valuable suggestions for designing and fabricating high-performance ADC. In Section 2, the ADCs, as shown in Figure 2, including successive approximate register (SAR) ADC, sigma-delta (Σ-∆) ADC, pipelined ADC and hybrid ADC, are systematically introduced. ADCs with different bandwidths and resolution performances can be utilized in different ultrasonic imaging systems. The performance indexes are compared and discussed in Section 3. Finally, the summarization and outlooks of ADC for miniatured UIS are presented.

## 2. ADC for Miniaturized High-Resolution UIS

The ADC, a bridge between the analog world and the digital world, converts the analog signals to digital so that the analog signal in the baseband can be processed by the back-end computer or microprocessor. The accuracy, speed, and linearity of ADC have an important influence on the quality of ultrasonic imaging, and generally, ADC with above 12-bit resolution and tens of MHz sampling rate could meet the performance requirement of traditional UIS. However, higher resolution and speed are desirable for the developed UIS, such as the color Doppler imaging, 3D imaging, and elastography systems.

### 2.1. SAR ADC

**Figure 3:**
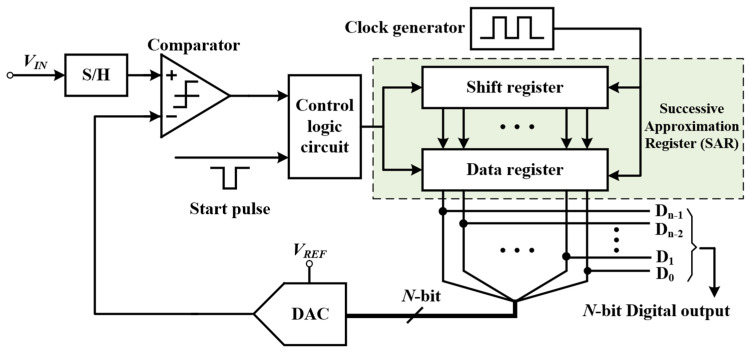
Basic structure diagram of SAR ADC.

The SAR ADC, shown in Figure 3, is a data converter with medium speed and accuracy. The core circuit is generally composed of digital control logic, sample-and-hold circuit (S/H), N-bit register, comparator, and N-bit DAC. Its working process is as follows: the highest bit of the shift register is set as 1, instructing that the DAC outputs the corresponding voltage to the inverting terminal of the comparator for comparison with the input V_IN_. If V_IN_ is greater than this voltage, the comparator outputs 1, and then the shift register samples and saves the highest bit as 1. Otherwise, it is 0. Until the last bit is compared, the register locks the data and outputs them. Due to adoption of the binary search algorithm, SAR ADC does not need additional coding circuits. In addition, as only one comparator is used in the circuit, it makes the circuit complexity increase slowly with the number of bits, and therefore power consumption and occupied area will be very low. The SAR ADC has become one of the choices of UIS requiring low power and small area A/D convertor.

**Figure 4:**
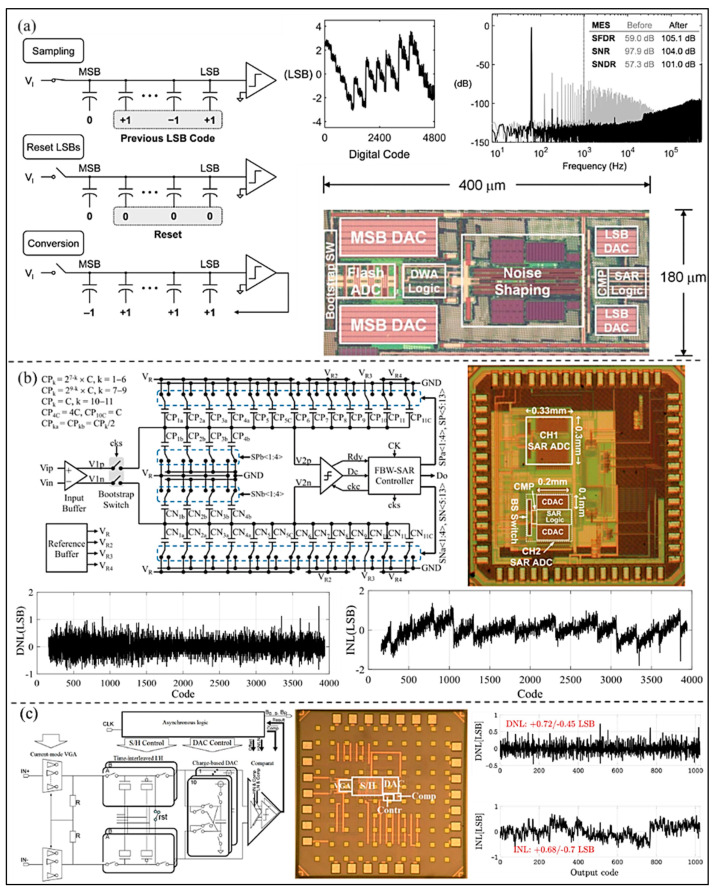
(**a**) SAR ADC operation with MES, simulated output spectrum, and chip photograph of 12-bit SAR ADC. (Reprinted from [35], Copyright 2016, with permission from IEEE); (**b**) Circuit architecture of the proposed FBW-SAR ADC, die photograph, and the measured performance. (Reprinted from [36], Copyright 2018, with permission from IEEE); (**c**) Top-level VGA/ADC architecture, die photo, and the experimental result. (Reprinted from [37], Copyright 2012, with permission from IEEE).

Although the SAR ADC has the advantages noted above, linearity performance of the SAR ADC is not excellent, which may finally deteriorate the quality of UIS imaging. Shu et al. [35] proposed a SAR ADC by using a DAC mismatch error shaping (MES) technique with oversampling to improve the linearity performance of the overall ADC, as shown in Figure 4a. Experimental results show that the linearity featured by spurious free dynamic range (SFDR) of the SAR ADC is over 100 dB with MES. Combined with a noise shaping scheme, the developed SAR ADC can reach >100 dB signal-to-noise plus distortion ratio (SNDR) without the need for factory trimming or digital calibration. The integral nonlinearity (INL) is within about +3/−3 LSB. In addition, a 12 bit 40 MS/s SAR ADC (as shown in Figure 4b) realized fast-binary-window (FBW) DAC switching technology was proposed by Chung et al. [36]. The bit conversion glitch generated by the underestimated routing parasitic capacitance and reference errors can be eliminated by the FBW DAC switching technology. Linearity of the developed ADC is improved without increasing the input capacitance or the use of calibration scheme. The peak differential nonlinearity (DNL) and INL are +1.47/−0.86 and +1.3/−1.8 LSB, respectively. Moreover, in order to improve the linearity of SAR ADC, Malki et al. [37] designed a SAR ADC with adaptive dynamic range (DR), as shown in Figure 4c. The developed SAR ADC is combined with a current steering variable gain amplifier (VGA) to avoid the need for a high-power buffer in front of the ADC. The non-linear voltage characteristics of metal-oxide-semiconductor (MOS) capacitors are solved by using a charge-domain ADC. For a sampling rate of 40 MS/s, the linearity performance is DNL within +0.72/−0.45 LSB, and INL +0.68/−0.7 LSB.

**Figure 5:**
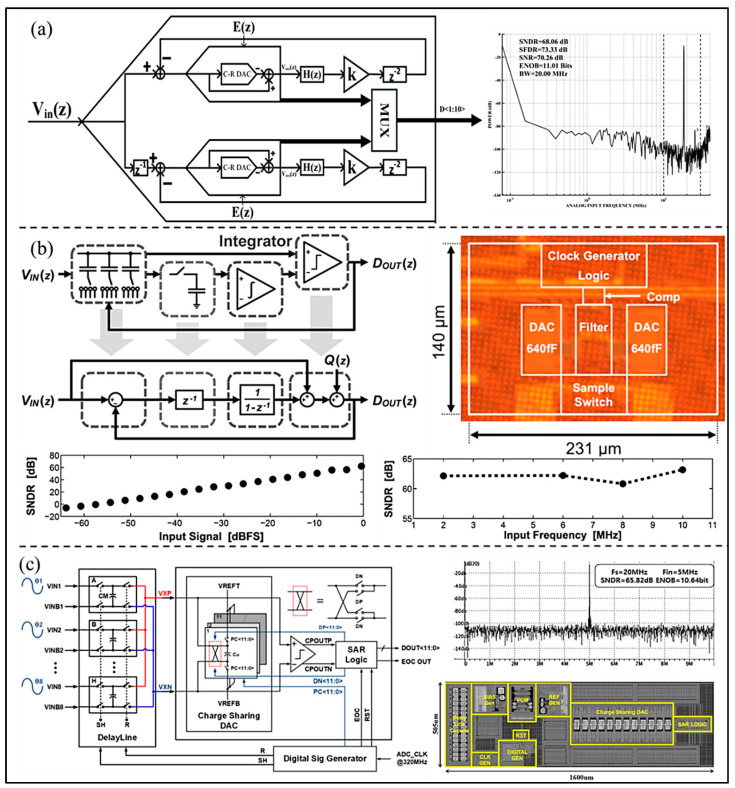
(**a**) Basic structure and simulation spectrum of proposed band-pass noise-shaping ADC. (Reprinted from [38], Copyright 2019, with permission from IEEE); (**b**) Circuit structure, measured performance, and die photograph of the proposed ADC. (Reprinted from [39], Copyright 2012, with permission from IEEE); (**c**) Block Diagram, simulation results, and layout of the proposed SAR ADC. (Reprinted from [40], Copyright 2021, with permission from IEEE).

To achieve better noise suppression and therefore increase the resolution, Li et al. [38] proposed a SAR ADC based on time interleaving, as shown in Figure 5a. Compared with fully-passive noise shaping SAR ADC, the total capacitance in the circuit is reduced by about four times because a high efficiency operational amplifier with a shared switched capacitor filter is used to replace the capacitors. The experimental results show that the ADC achieves a SFDR 73.33 dB and an effective number of bits (ENOB) 11-bit in a bandwidth of 20 MHz. Fredenburg et al. [39] proposed a noise shaping SAR ADC using the technology of decoupling the accuracy of the comparator from the resolution of ADC, as shown in Figure 5b. The DAC residue voltage produced at the end of the SAR conversion is used to perform noise shaping. An integrator is inserted between the passive sampling network and the inverting terminal of the comparator to improve the resolution gain. The circuit then behaves exactly like a first-order Σ-Δ modulator. Even if the integrator quality is low, it can achieve good noise suppression. The ADC was fabricated in a 65 nm CMOS process and occupies an area of only 0.03 mm^2^. The signal bandwidth is 11 MHz, and it consumes 806 μW from a 1.2 V power supply. The measured SFDR is 72 dB, and ENOB 10-bit, and with a 2 MHz input signal sampled at 90 MS/s. Moreover, in order to decrease the noise, area, and power, Lee et al. [40] proposed a charge sharing SAR ADC for ultrasound diagnostic medical devices (as shown in Figure 5c), which includes delay line circuits, a charge sharing DAC, a comparator, an SAR logic, and a digital signal generator. The developed 12-bit ADC fabricated in 130 nm CMOS process occupies 1600 × 505 μm, and the sampling rate is 20 MS/s. The simulation results show that the proposed ADC can achieve an ENOB of 10.64-bit, SNR of 70.06 dB, and SNDR of 65.82 dB at the input frequency of 5 MHz.

**Figure 6:**
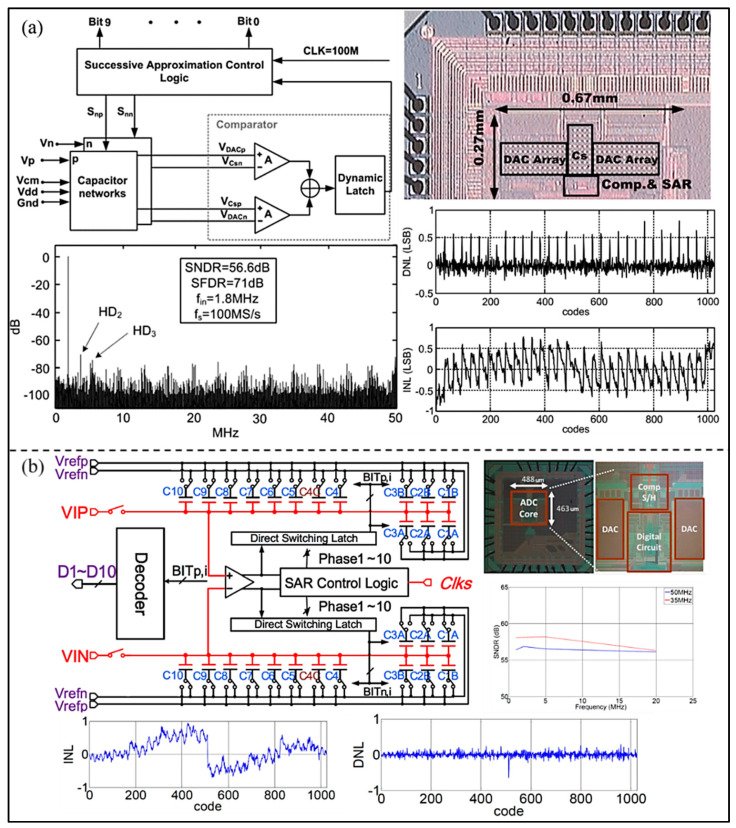
(**a**) Overall schematic diagram, die microphotograph, and the digital output FFT of the ADC. (Reprinted from [41], Copyright 2010, with permission from IEEE); (**b**) Architecture, die microphotograph, and measured results of the proposed SAR ADC. (Reprinted from [43], Copyright 2015, with permission from IEEE).

In order to reduce power consumption and improve conversion linearity, Zhu et al. [41] proposed a SAR ADC by using no-reference technology, which can avoid the static power consumption of the on-chip reference generator and realize the high-speed and low-power operation. In addition, due to the adoption of a common-mode-based charge recovery switching method, the switching energy is reduced and the conversion linearity is improved. As shown in Figure 6a, the power consumption is 3 mW with a voltage supply of 1.2 V, the peak SNDR 56.6 dB, and SFDR 71dB at a 100 MS/s sampling rate. Xu et al. [42] proposed a 7-bit 50 MS/s SAR ADC used in UIS to decrease the power and area. The proposed SAR ADC adopts a single-ended architecture using the double references technique. The ENOB, area, and power consumption of the proposed SAR ADC are 6.5 bits, 0.017 mm^2^, and 157 μW, respectively.

**Figure 7:**
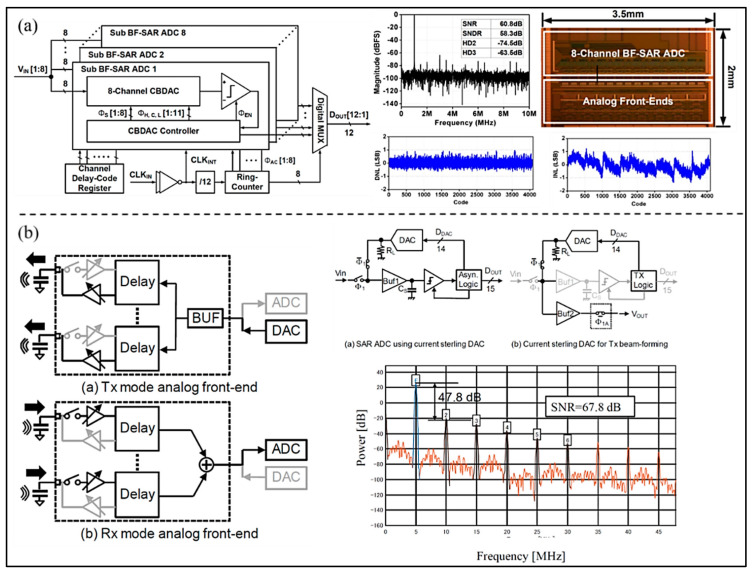
(**a**) Block diagram, die micrograph, and measured results of the proposed SAR ADC. (Reprinted from [45], Copyright 2017, with permission from IEEE); (**b**) Structure diagram and measured results of SAR ADC. (Reprinted from [46], Copyright 2018, with permission from IEEE).

To extend the battery life of a portable ultrasonic system, Tsai et al. [43] presented a 10-bit low-voltage SAR ADC, based on a 180 nm manufacturing process, without an additional level shifter for shrinking the input signals (as shown in Figure 6b). It operates at dual-voltage domain and minimizes the use of a 1.8 V supply voltage and 3.3-V I/O devices, occupying an active area of 0.226 mm^2^. The results show that ENOB and power consumption of the proposed ADC are 9.15 bits and 1.6 mW at 50 MS/s. In addition, Chen et al. [44] proposed a front-end application-specific integrated circuit (ASIC) for miniature 3D ultrasound probes utilizing a low-power charge-domain SAR ADC. The adopted ADC merges directly with the sample-and-hold delay lines in each subarray and employs high-speed data links around the ASIC, so that channel count reduction can be achieved. The measured results show that the ADC adopted in design can achieve a sampling rate of 33 MHz, and the developed ASIC consumes 130.7 mW at a 1.8 V power supply. Kim et al. [45] proposed an analog beamforming embedded SAR (BF-SAR) ADC for UIS, which consists of multiple sub-beamforming SAR ADCs (as shown in Figure 7a). As the ADCs complete analog beamforming and A/D conversion sequentially without a summing op-amp, power consumption can be saved observably, and it is only 12.1 mW. The proposed BF-SAR ADC is sampled at a frequency of 20 MHz and achieves a SNR of 60 dB. The peak DNL and INL are 0.97/−0.67 LSB and 1.24/−1.37 LSB, respectively. Moreover, to reduce power consumption, Yazaki et al. [46] presented a new circuit structure of a 12-bit SAR ADC used for reception of the ultrasonic diagnostic apparatus (as shown in Figure 7b). A DAC is applied in the SAR ADC, which is time-shared by Tx and Rx beamforming. The developed circuit can achieve beamforming without adding circuits in Tx mode. Simulation results show that the SNR is 67.8 dB and the second harmonic distortion is 47.8 dB at a sampling rate of 160 MHz.

### 2.2. Sigma- Delta ADC

**Figure 8:**
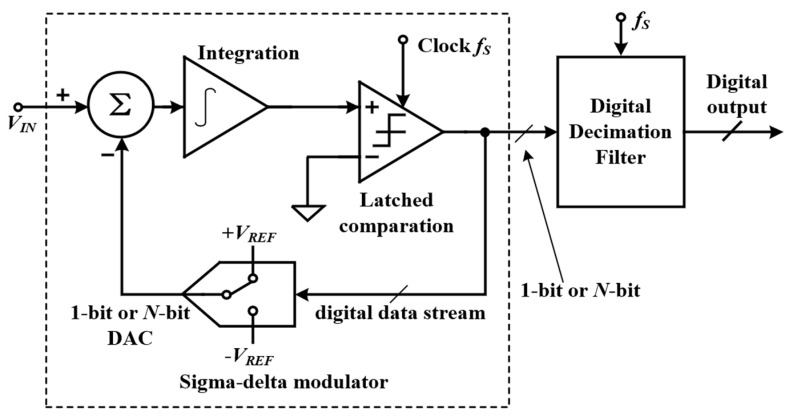
Basic structure diagram of Σ-∆ ADC.

A typical Σ-∆ ADC architecture consists of an analog Σ-∆ modulator (SDM) and a digital decimation filter. The analog SDM can shape the quantization noise and move it to the high frequency domain, and the digital decimation filter, which is implemented by digital circuits, then removes the high frequency noise components. As shown in Figure 8, the working principle of Σ-∆ ADC is: first, the input signal and the output signal of 1-bit or multi-bit DAC are differentiated (represented by ∆) to obtain an analog differential signal, and then the signal is integrated (represented by Σ for integration). After that, the signal is compared with the reference voltage to obtain a 1-bit or multi-bit ADC output. In the low-frequency field, due to the employing of oversampling and noise shaping, Σ-∆ ADC can achieve very high resolution, but with a relatively small bandwidth. These characteristics make it suitable for accurate measurement field and high-resolution ultrasonic imaging. Since the power consumption is directly proportional to the sampling rate, a compromise among the oversampling ratio, SNDR, and power consumption of the Σ-∆ ADC should be considered carefully.

**Figure 9:**
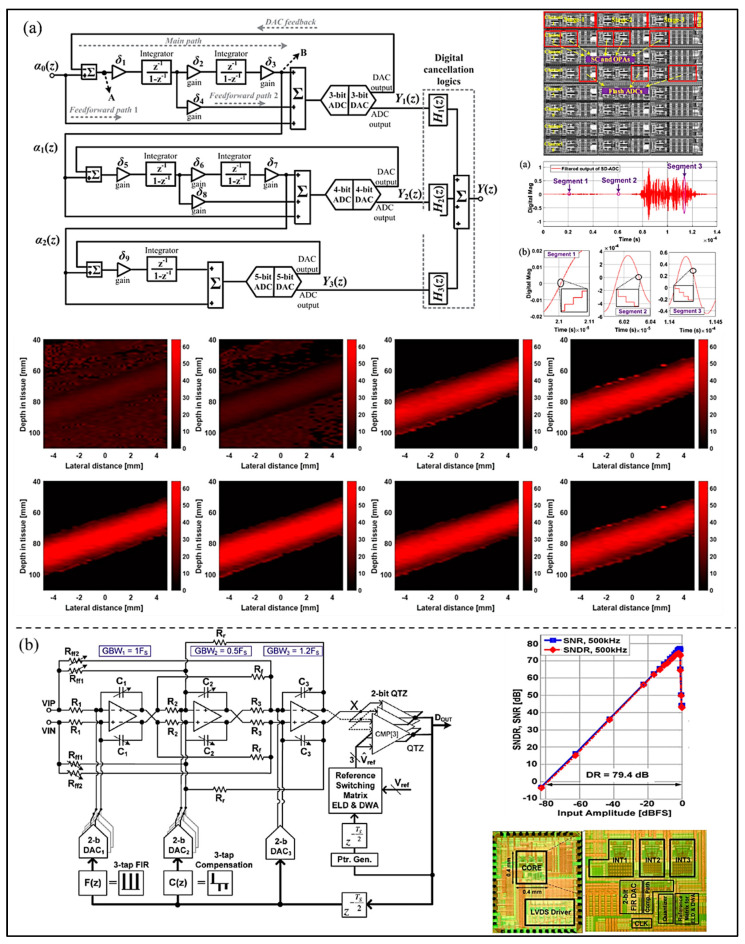
(**a**) Schematic of the proposed MASH Σ-∆ modulator, die microphotograph, and the simulated performance. (Reprinted from [47], Copyright 2020, with permission from IEEE); (**b**) Schematic of the overall CT Σ-∆ modulator, measured result, die microphotograph, and 2D ultrasonic doppler images. (Reprinted from [48], Copyright 2015, with permission from IEEE).

To further improve the resolution, Li et al. [47] proposed a high-resolution ADC using multi-stage noise shaping (MASH) Σ-∆ modulator for color Doppler imaging. The quantization noise is shaped by a MASH2_3b_-2_4b_-1_5b_ Σ-∆ modulator, and then the shaping noise is filtered by a digital decimation filter at high frequency. As shown in Figure 9a, the circuit was designed in 0.35 μm CMOS technology, and the SNDR was 109.5 dB, the ENOB 17.9-bit in a bandwidth of 8 MHz. The results show that the proposed Σ-∆ ADC provides superior performance on image estimation accuracy and contrast of the blood speed. It lays a foundation for the development of UIS and color Doppler imaging systems to a large extent. Furthermore, Zhang et al. [48] proposed a continuous-time (CT) Σ-∆ modulator fabricated in 65 nm CMOS process. The delay free feedback path can be achieved by the combination of a digitally controlled reference switch matrix, excess loop delay, and data weighted average (DWA) technology. In addition, the lower clock jitter sensitivity and better loop filter linearity can be realized due to the multi-bit FIR feedback DAC and its compensation path. As shown in Figure 9b, the SNR of the proposed modulator is 77.3 dB in a 15 MHz bandwidth, with SNDR 74.3 dB.

**Figure 10:**
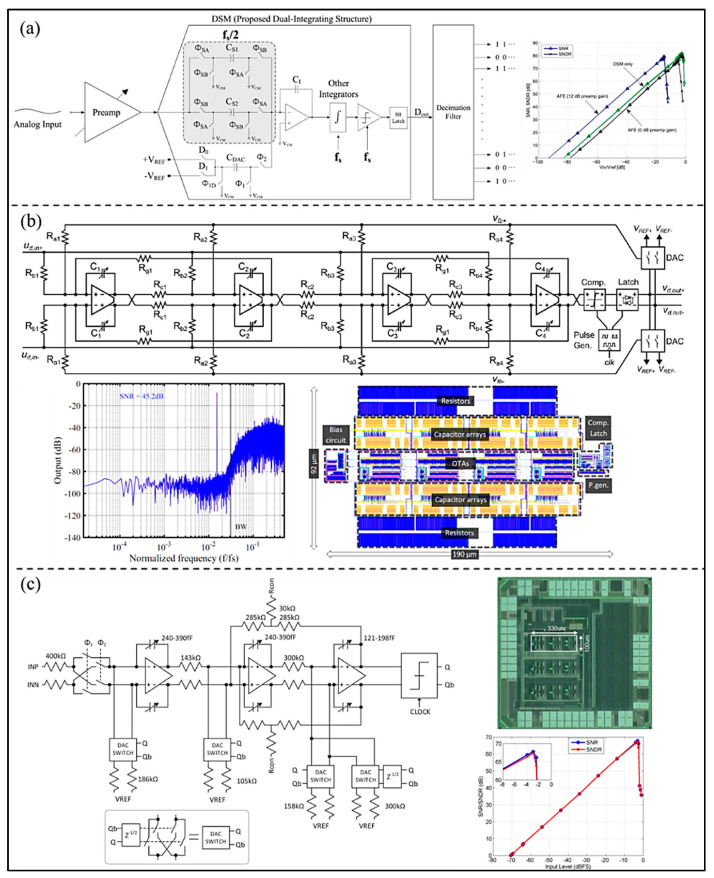
(**a**) Structure and measurement result of the proposed DI-DSM. (Reprinted from [49], Copyright 2016, with permission from IEEE); (**b**) Structure, simulated frequency response, and layout of the CTDS ADC designed. Reprinted from [50], Copyright 2017, with permission from Springer Science and Business Media, LLC); (**c**) Simplified schematic representation, measured result, and die photo of the implemented modulator. (Reprinted from [51], Copyright 2016, with permission from IEEE).

To supply more sufficient and stable time for the key blocks, Yoon et al. [49] proposed a high-resolution dual-integrating delta–sigma modulator (DI-DSM) adopting dual input paths with slower clocks (as shown in Figure 10a). The feedback DAC of integrator works on conventional fast clocks to eliminate the possibility of noise folding and decrease the area. The results show that the peak values of SNR and SNDR are 81.4 dB and 80.4 dB, respectively, and the power consumption is 24.8 µW at a 1 V power supply voltage and 10 kHz bandwidth. In order to satisfy the portable requirement of UIS and decrease the power consumption, Muntal et al. [50] proposed a fully differential fourth-order 1-bit CT Σ-∆ ADC used for a portable ultrasound scanner, as shown in Figure 10b. The power consumption measured on the fabricated IC designed in a 65 nm process is 594 μW, and the SNR is 45.2 dB within a normal bandwidth 6.4 MHz. In addition, to decrease the power consumption, Kaald et al. [51] developed a single-bit third-order feedback type CT Σ-∆ modulator with non-return-to-zero DAC, as shown in Figure 10c. Each modulator occupies an active area of 0.033 mm^2^. The optimization methodology used in the ADC is the combination of a simulated annealing framework and a C++ simulator. The SNDR of CT-DSM reaches 67.4 dB in 1 MHz bandwidth, and its power consumption is only 131 μW, which verifies the high power-efficiency of the Σ-∆ modulator.

**Figure 11:**
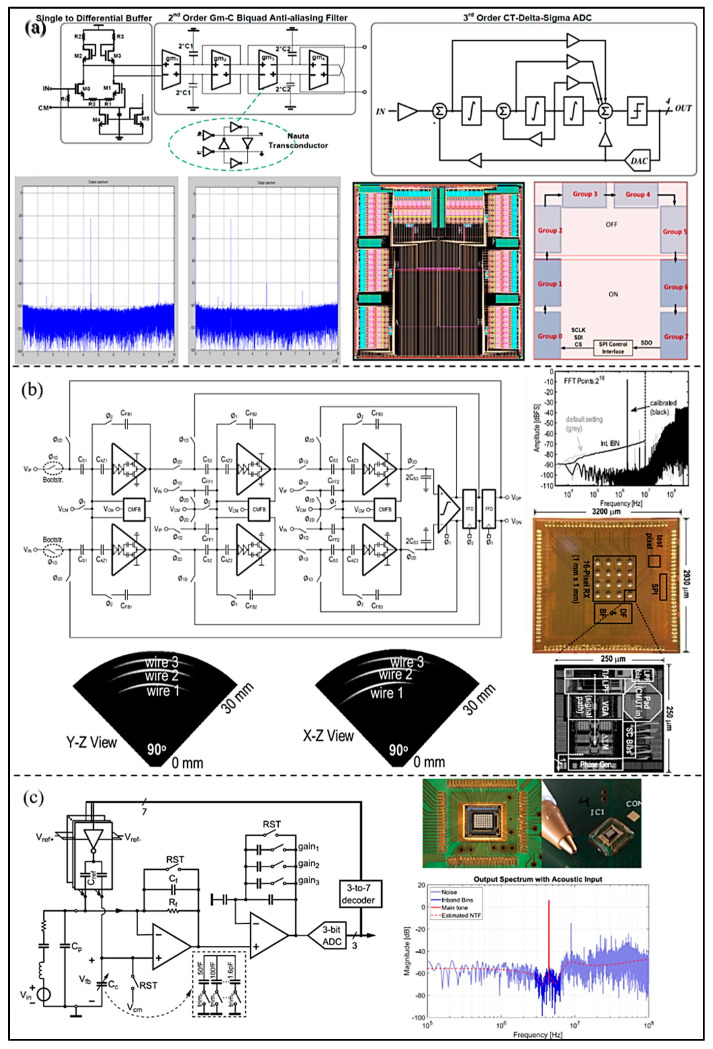
(**a**) IC architecture, layout, and measured results of the ADC in the design. (Reprinted from [52], Copyright 2015, with permission from IEEE); (**b**) Circuit implementation, die microphotograph, and measured results of the proposed ΔΣ ADC. (Reprinted from [53], Copyright 2017, with permission from IEEE); (**c**) Implemented ADC architecture, die microphotograph, and measured results. (Reprinted from [54], Copyright 2018, with permission from IEEE).

For a handheld wireless UIS, Chirala et al. [52] introduced a 128-channel CT ∆-Σ ADC based mixed signal chipset fabricated in 0.13 μm CMOS process (as shown in Figure 11a). The measured SNR of the ADC is about 65 dB, the ENOB 10.57 bits, and the power consumption 36.1 mW/channel. Because Nyquist ADC consumes more chip area while implemented in the system, Chen et al. [53] proposed a pixel pitch-matched ultrasonic receiver with Σ-∆ beamformer, used for 3D photoacoustic imaging (as shown in Figure 11b). The proposed Σ-∆ modulator adopts slewing-dominated and area-optimized inverter-based amplifiers. The SNDR and power consumption are 59.9 dB and 6.65 mW at 10 MHz bandwidth. Moreover, to simplify the receiver chain and save area and power consumption, an element-level CT ∆-Σ ADC for miniature endoscopic probes of 3D cardiac imaging (as shown in Figure 11c) was presented by D’Urbino et al. [54]. In the proposed architecture, a piezo-electric transducer element as a signal source is used as the electromechanical loop filter of a bandpass Σ-∆ modulator for decreasing amount of circuitry. The measured results show that the proposed ADC achieves a SNR of 47 dB and the power consumption is 800 μW at a 1.8 V supply.

### 2.3. Pipelined ADC

**Figure 12:**
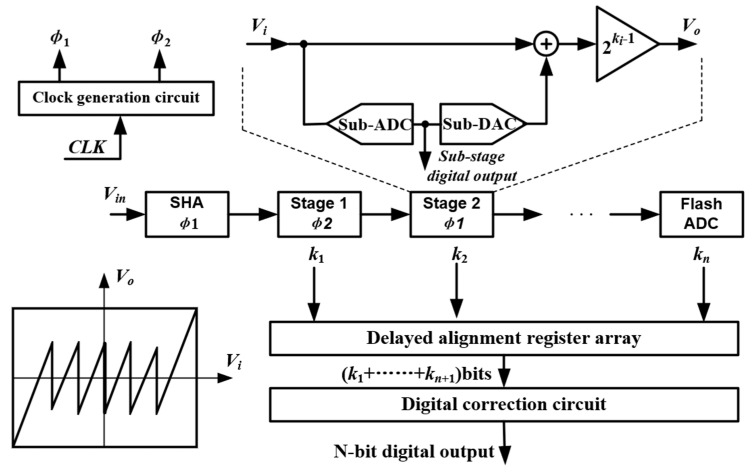
Basic structure diagram of pipelined ADC.

The pipelined ADC, which is controlled by the two-phase non-overlapping clock, can be understood as a multi-step ADC. Each conversion stage has two working states: the sampling state, and the amplifying and holding state. The working principle of pipelined ADC is as shown in Figure 12: first, the input signal is sampled by a sample/hold amplifier (SHA) circuit and then sent to the pipelined conversion stage where the signal is quantized step by step. The digital correction circuit is used for delay alignment afterwards, and finally an N-bit digital code is obtained. Each sub-stage in the pipelined ADC can work in parallel, and thus the conversion rate of the pipelined ADC can be very high. However, employing of many sub-stages to meet high resolution requirement will certainly lead to higher circuit complexity, and therefore high-power consumption and large occupied area.

**Figure 13:**
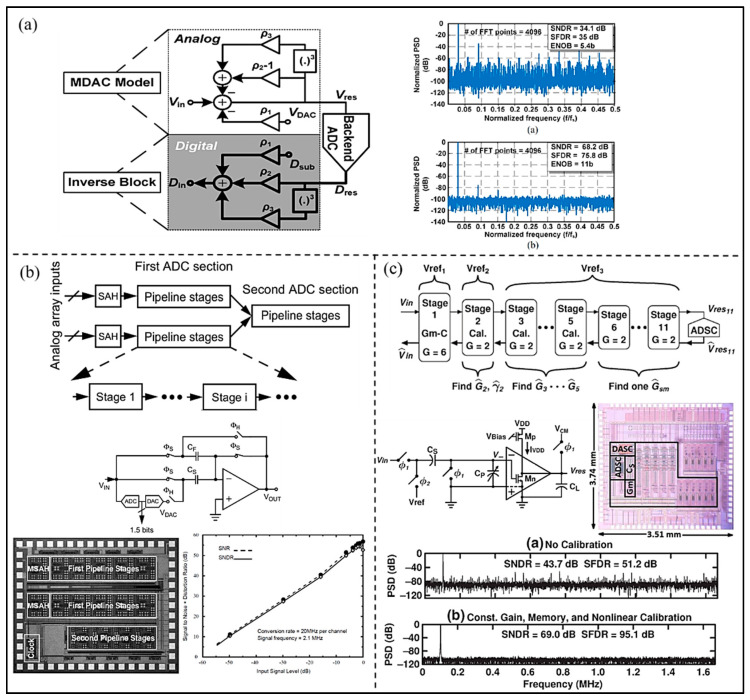
(**a**) Calibration procedure and simulated ADC output spectrum. (Reprinted from [55], Copyright 2017, with permission from IEEE); (**b**) Architecture and die photo of the proposed ADC and measured result. (Reprinted from [56], Copyright 2003, with permission from IEEE); (**c**) Block diagram of the prototype, schematic of a gm-C-based RA, the output spectra of the developed ADC, and die photo. (Reprinted from [57], Copyright 2015, with permission from IEEE).

In order to improve the accuracy of pipelined ADC, Gholami et al. [55] proposed a digital background calibration technology called decision point histogram (DPH) correction (as shown in Figure 13a). The capacitor mismatch and residual amplifier gain error can be corrected by estimating the output codes of the decision points in the residual characteristics. The results show that the SNDR of the developed ADC is improved from 34.1 dB to 68.2 dB, and SFDR 35 dB to 75.8 dB. In addition, to optimize the performance of ultrasound imaging, the performance and channel number of the ADC for UIS are required to be improved. To reduce the area of the converter, Kaviani et al. [56] proposed an 8-channel 10-bit pipelined ADC using multiplexing technology for UIS. The modular architecture provides a practical option for array processing and allows the integration of larger sensor arrays as the technology shrinks to smaller feature sizes. As shown in Figure 13b, the peak SNR and SNDR of the proposed pipelined ADC are 54.3 dB and 56.8 dB, respectively. The prototype chip occupies less than 4 mm^2^ of silicon area and dissipates a total power of 330 mW from a 2.5 V supply. To improve SNR of ADC, Wang et al. [57] proposed a 12-bit pipelined ADC using an open-loop residual amplifier based on the first stage integrator (as shown in Figure 13c). In the developed ADC, the first three ADC stages are supplied by separate reference voltages to reduce interstage coupling. The SFDR of the ADC is improved from 51.2 dB to 95.1 dB, and SNDR from 43.7 dB to 69.0 dB.

**Figure 14:**
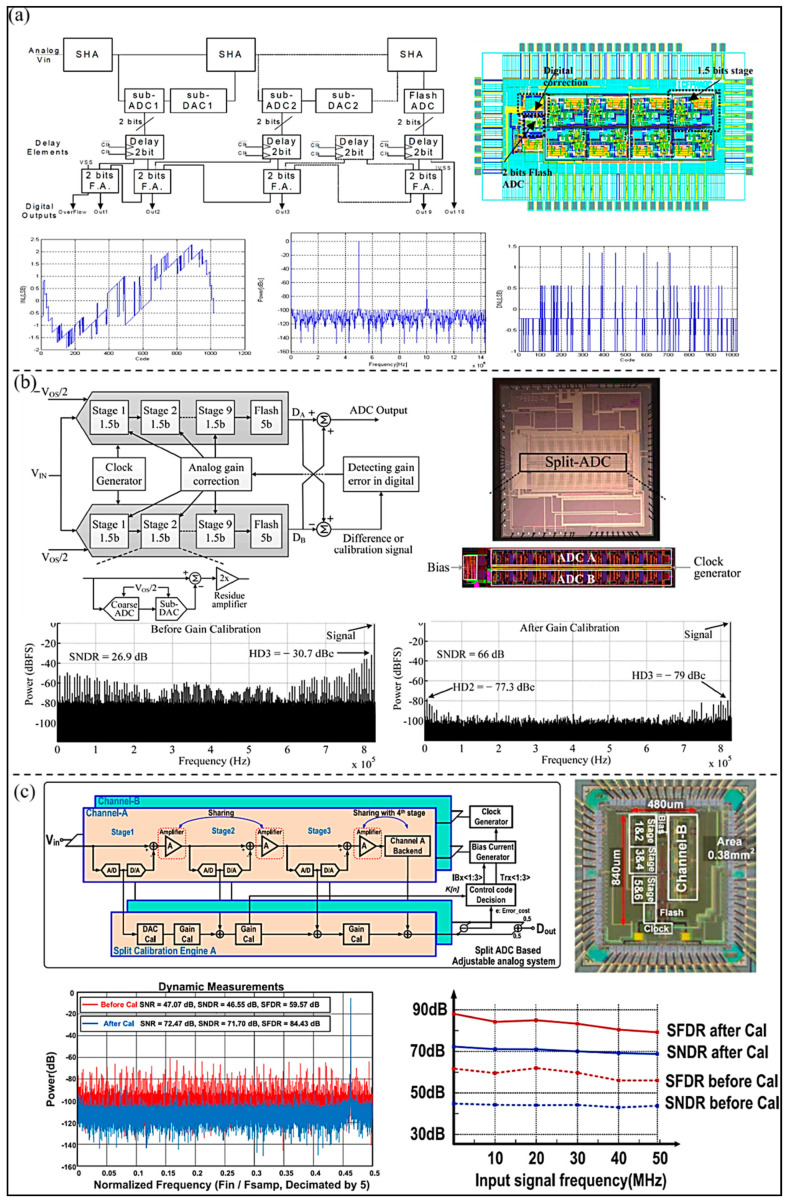
(**a**) Block diagram, layout, and measured results of the proposed ADC. (Reprinted from [58], Copyright 2002, with permission from IEEE); (**b**) Structure of the implemented pipelined split-ADC, die photograph, and experimental results. (Reprinted from [59], Copyright 2018, with permission from IEEE); (**c**) Block diagram and chip micrograph of the split-pipelined ADC with digital calibration and the measured result. (Reprinted from [60], Copyright 2018, with permission from IEEE).

In order to achieve low power consumption and small area, El-Sankary et al. [58] proposed a low voltage 10-bit pipelined ADC for ultrasonic receivers (as shown in Figure 14a). An enhanced fully differential telescopic operational amplifier with cascade code compensation is adopted in the design to meet the 10-bit ADC accuracy at 50 MS/s sampling frequency. By using a dynamic comparator and careful optimization of the different blocks of the proposed ADC, power consumption is reduced to 31 mW. The developed ADC can achieve a SNR of 61 dB at 5 MHz, and the measured DNL and INL are 1.35 LSB and 2.25 LSB, respectively. In addition, in order to improve power efficiency, Akter et al. [59] proposed a split-pipelined ADC with a closed-loop class AB residual amplifier (as shown in Figure 14b). The overall ADC is composed of nine substages (1.5-bit per stage) and a 5-bit flash ADC. The class AB residual amplifier consists of a push-pull structure and a ‘split capacitor’ bias circuit. The proposed ADC dissipates 9 mW, and experimental results show that SNDR 66 dB and SFDR 77.3 dB are achieved. Moreover, Mao et al. [60] proposed a 14-bit split-pipelined ADC to improve the performance of pipelined ADC in power efficiency (as shown in Figure 14c). The proposed ADC employs background calibration and can optimize the amplifier power consumption in the shared operational amplifier. Due to the calibration, the peak SNDR and SFDR of the proposed ADC are improved to 71.7 dB and 84.4 dB, respectively. It can maintain an SNDR over 68.5 dB within the full Nyquist bandwidth, consuming 32 mW of power.

**Figure 15:**
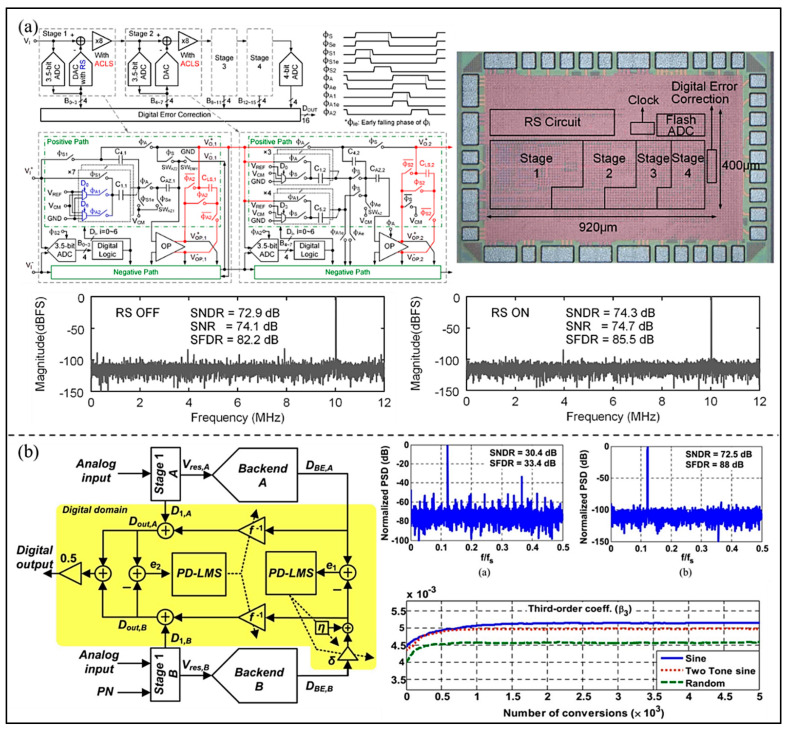
(**a**) Circuit architecture, chip micrograph, and measured spectra of the proposed ADC. (Reprinted from [61], Copyright 2019, with permission from IEEE); (**b**) The proposed digital background calibration and simulated spectrum. (Reprinted from [62], Copyright 2015, with permission from IEEE).

The accuracy and linearity of the ADC can be greatly deteriorated by various non-ideal errors, especially the mismatch in DAC elements and gain errors. In order to reduce or suppress these nonlinearities, Hung et al. [61] proposed a pipelined ADC adopted reference swapping (RS) technology. By using the averaging operation and the combination with a simple capacitor layout arrangement, the RS technology reduces the capacitor random mismatch error and capacitor gradient mismatch error. The results show that the proposed pipelined ADC achieves 74.3 dB SNDR and 85.5 dB SFDR (as shown in Figure 15a), and power consumption is 5.1 mW. Moreover, to correct the gain error in substages for pipelined ADC, Montazerolghaem et al. [62] proposed a predetermined least mean square (LMS) digital background calibration technology (as shown in Figure 15b). In this method, the pipelined ADC is divided into two equal channels, which can perform digital background calibration by using the pseudo-random sequence to change the decision point of the sub-ADC. Through simulation experiments, the convergence time of the proposed LMS calibration technique is significantly reduced compared with the traditional LMS algorithm. In addition, the peak SNDR and SFDR of the proposed ADC are improved to 72.5 dB and 88 dB due to the calibration.

### 2.4. Hybrid ADC

SAR ADC has great advantages in terms of power consumption and low circuit complexity. However, only low-to-medium precision and speed can be achieved because it is quantized in a step-by-step comparison scheme. The Σ-∆ ADC can achieve very high accuracy due to the use of noise shaping and oversampling technology, whereas it is generally slower and can be only used in high-precision and low-speed applications. The developed pipelined ADC can achieve medium precision and high speed, but the use of the excessive number of sub-stages will inevitably result in circuit complexity and high-power consumption. The hybrid ADC is proposed in recent years considering the performance limitations of SAR, Σ-∆ and pipelined ADCs. By combining two or more types of ADCs, performance of the A/D conversion can be improved, and therefore so can the imaging quality of the ultrasound imaging system.

**Figure 16:**
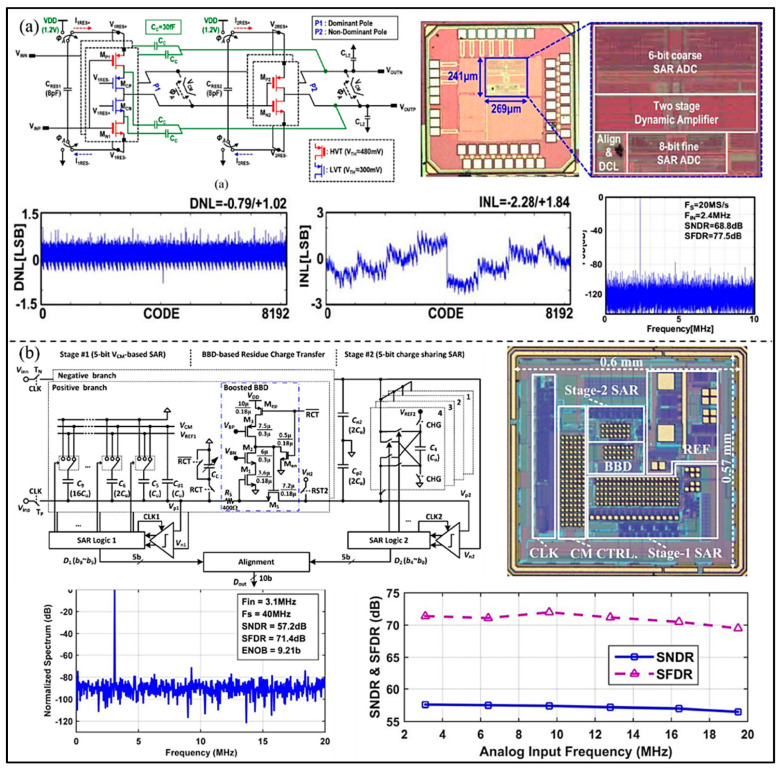
(**a**) The proposed chip microphotograph and frequency response comparison of ADC. (Reprinted from [63], Copyright 2021, with permission from IEEE); (**b**) Architecture of the proposed ADC, die microphotograph, and the measured performance. (Reprinted from [64], Copyright 2018, with permission from IEEE).

In order to make better use of the advantages of each type of ADC, the hybrid ADCs have been proposed and continuously developed. Kwon et al. [63] proposed a closed-loop two-stage dynamic amplifier for pipelined-SAR ADC. The proposed two-stage dynamic amplifier has a high DC gain, and good robustness against process, voltage, and temperature (PVT) variations without further calibration (as shown in Figure 16a). The developed pipelined SAR ADC can achieve a SNDR of 68.8 dB and maintain SNDRs over various sampling rates from 1 to 20 MS/s. The power consumption is only 348 µW from a 1.2 V power supply. To reduce power consumption and improve accuracy, Zhang et al. [64] proposed a low-power pipelined-SAR ADC, which adopts the booster barrel brigade device (BBD) to deal with the residual charge, as shown in Figure 16b. However, because the boost BBD introduces large nonlinearity and serious cumulative common mode charge error, a real-time calibration circuit with high power consumption is needed. The SNDR and SFDR of the proposed ADC are 57.1 dB and 71.4 dB, and the power consumption is 1.87 mW.

**Figure 17:**
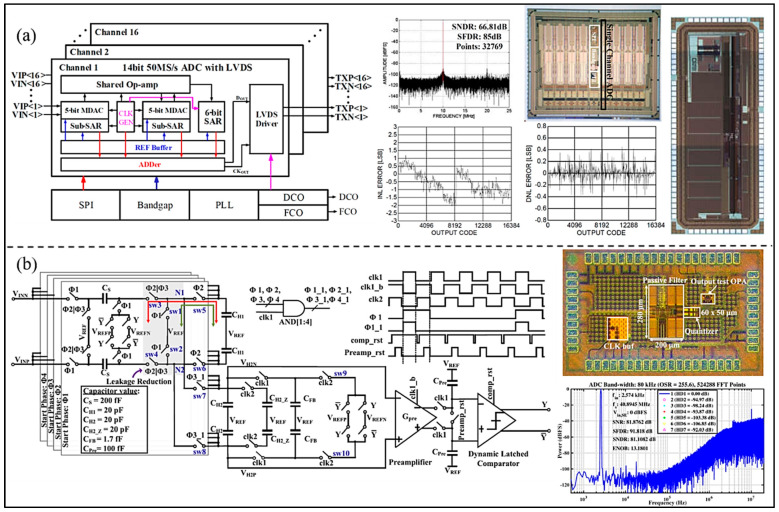
(**a**) Block diagram, layout, and measured results of the proposed ADC. (Reprinted from [65], Copyright 2020, with permission from IEEE); (**b**) Circuit architecture, timing diagram, die, and measured result of the developed ADC. (Reprinted from [66], Copyright 2020, with permission from IEEE).

To achieve the best power and area efficiency, Wu et al. [65] presented a 16-channel 14-bit pipelined-SAR ADC for integrated UIS implemented in a 0.18μm process (as shown in Figure 17a). The ADC, designed with complete peripheral circuits including low voltage differential signaling (LVDS), serial peripheral interface (SPI), bandgap, etc., occupies an area of 0.625 mm^2^ and achieves a peak SFDR of 85.6 dB, a SNDR of 66.8 dB at 50 MS/s sampling rate. The maxima of DNL and INL are −0.46/0.6 LSB and −2.53/1.32 LSB, respectively. In addition, in order to improve the noise suppression and stability of the modulator, Wang et al. [66] proposed the passive switched-capacitor (SC) Σ-∆ modulator based on pipelined structure (as shown in Figure 17b). The charge-sharing rotation technique is adopted for the proposed modulator, which can eliminate any inter-stage loading effects that plague conventional SC passive modulators. The proposed ADC was fabricated in a 28 nm CMOS process and achieves a SNDR of 81.1 dB. The power consumption is 101.5 μW from a 1 V power supply.

**Figure 18:**
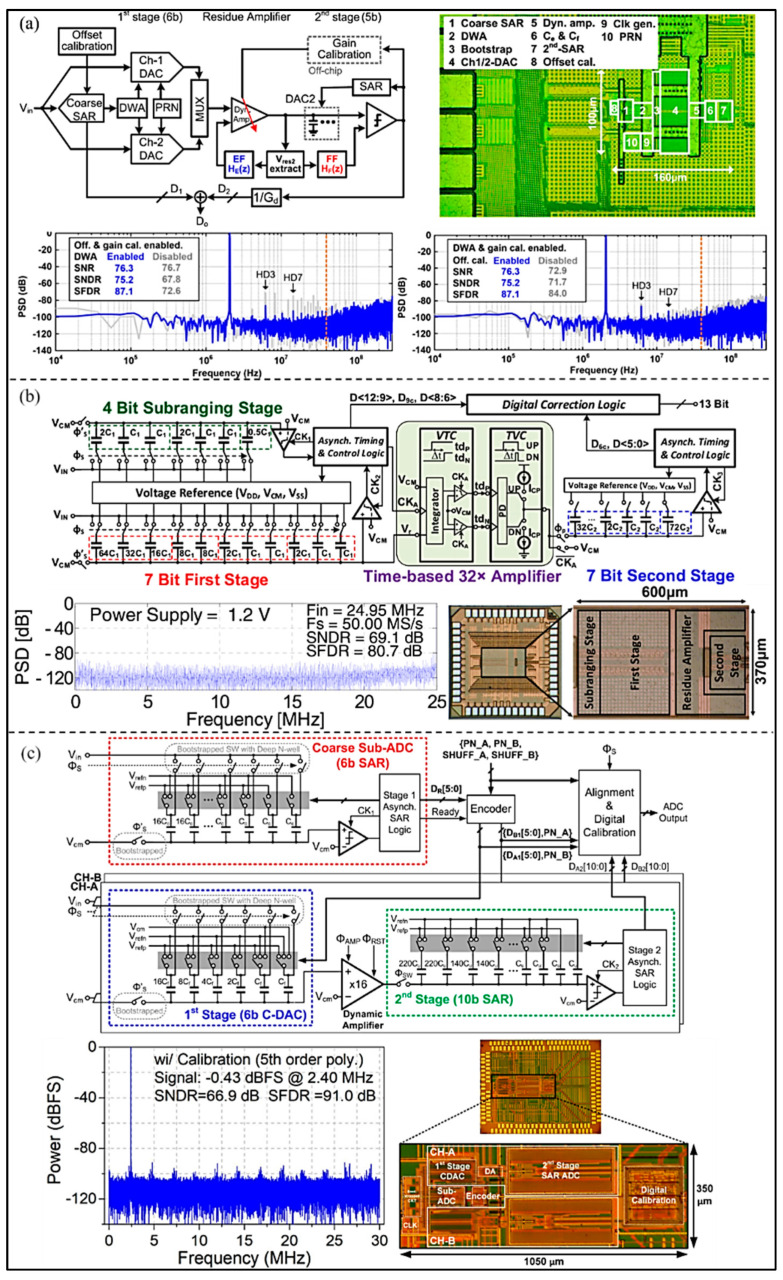
(**a**) Block diagram, layout, and measured results of the proposed ADC. (Reprinted from [67], Copyright 2020, with permission from IEEE); (**b**) Circuit architecture, timing diagram, die, and measured result of the developed ADC. (Reprinted from [68], Copyright 2017, with permission from IEEE); (**c**) Block diagram, layout, and measured results of the proposed ADC. (Reprinted from [69], Copyright 2020, with permission from IEEE).

Speed and accuracy are the two most important performance parameters for an ADC. In order to improve the overall speed and sampling performance of ADC, Song et al. [67] proposed a partial-interleaving SAR-assisted noise-shaping pipelined ADC (as shown in Figure 18a). In the proposed ADC, a multi-input dynamic amplifier is adopted to achieve low-power first-order noise-shaping. In addition, the noise transfer function deterioration is compensated by using an additional residual feedforward path in the secondary SAR ADC. The SNDR of the hybrid ADC is 75.2 dB, whereas the over-sampling rate is only 7.5. The DWA, which is a first-order noise shaping algorithm, is used to improve the linearity SFDR from 72.6 dB to 87.1 dB. Moreover, Zhang et al. [68] proposed a 13-bit sub-ranging pipelined-SAR ADC (as shown in Figure 18b). It adopts a SAR-assisted sub-domain floating capacitive DAC switching algorithm, so that the linearity and speed are promoted and the switching energy is decreased. Temperature-insensitive residual amplifiers cannot only amplify the signal without background calibration, but also retain the advantages of dynamic operation and noise filtering. The prototype ADC, fabricated in a 130 nm CMOS process, can achieve a SNR of 69.1 dB and a SNDR 80.7 dB. In order to increase the conversion rate and also reduce power consumption, Cao et al. [69] proposed a single-coarse dual-fine pipelined-SAR ADC with split-based background calibration, as shown in Figure 18c. The shuffle mechanism, which can effectively avoid the divergence of the traditional algorithms in digital background correction based on split ADC, is employed in the architecture. The measurement results show that the SNDR and SFDR are significantly improved by the calibration, and they are 66.9 dB and 91.0 dB, respectively. The power consumption is 4.26 mW and the conversion rate is 60 MS/s.

## 3. Comparison and Discussion

The performance indexes of different ADCs, such as fabrication technology, bandwidth, power, area, INL and DNL, etc., are compared in Table 1. Generally, SAR ADC has lower power consumption and smaller area (see [35,37,41]); however, it cannot achieve high precision and high speed due to the limitation of the architecture. The ADC proposed in [35] can achieve the higher accuracy at the expense of reducing the sampling rate. The figure of merits (FoMs) of the ADCs in [35,37] are excellent due to the very low power consumption and high SNDR. Therefore, SAR ADC can be used to handle UIS, due to the advantages of small size and low power consumption.

In addition, Σ-∆ ADC usually can achieve ultra-high accuracy, which affects the imaging effect of UIS. The SNDR of the Σ-∆ ADC in [47], which is based on MASH structure, is the highest, thereby achieving the highest imaging effect. However, the bandwidth of Σ-∆ ADC is usually less than 10 MHz, which limits the application of Σ-∆ ADC. Because of the high accuracy of Σ-∆ ADC, it can be used in the high-precision portable UIS.

Moreover, pipelined ADCs in [56] and [59] have the characteristics of the higher speed and precision, but the power consumption is usually large. The choice of the fabrication process affects the area of the ADC circuit. Due to the adoption of the 250 nm manufacturing process, the ADC circuit in [56] takes up the largest area among the circuit structures listed in the table and its power consumption is 330 mW, which results in a very poor FoM of 4857 fJ/step. The pipelined ADC can be adopted in high-speed portable UIS because all conversion stages can operate simultaneously.

Compared with the above-noted ADCs, the hybrid ADC has the smaller area and higher speed. For example, the conversion speed of ADC in [68] is 50 MHz, the area of the ADC in [67] is 0.016 mm^2^. The FoM of the ADC in [67] is 0.9 fJ/step, as the power consumption is only 2.56 mW with a 600 MHz sampling rate. In addition, for work under different power supply voltages, a pipelined SAR ADC is proposed in [68], which has three working modes with different maximum conversion speeds. The hybrid ADC can be used in various portable UIS because it can achieve relatively good comprehensive performance due to the combination of two types of ADC.

## 4. Conclusions and Outlook

With the unique advantages of safety and operational flexibility, portable UIS has been widely used in medical imaging and diagnoses. The recent development of ADCs for miniaturized high resolution UIS is systematically introduced in this paper. Accuracy, linearity, and sampling rate of ADC have an important influence on the ultrasonic imaging quality. Different ADC structures have different advantages and shortcomings in resolution and speed. In addition, the hybrid ADC, which can achieve comprehensive performance, is introduced and compared with the general ADC. The introduction and comparison of these ADCs in this paper can provide valuable guidance to design high-performance ADC for miniaturized high resolution UIS.

With the requirements of small size, low-power, and high resolution portable UIS, the performance of ADC for portable UIS, such as noise, linearity, dynamic range, speed, resolution, chip area, and power consumption, should be improved in future research. The challenges of miniaturized high resolution UIS and the corresponding solutions are pointed out as follows:

1. The integration of ADC ICs should be improved.
  - Due to the compact size of capacitive micromachined ultrasonic transducer (CMUT) or piezoelectric micromachined ultrasonic transducer (PMUT) elements and the limited space available in the probe, efficient-area ICs design of the ADC, especially for multi-channel requirements, should be developed. Advanced fabrication process with smaller feature size is a fundamental way to improve the integration.
  - It is worth considering that the ADC ICs can be bonding integrated with CMUT chips at the wafer level through silicon via (TSV) technology, but the packaging problem should be solved.
  - The analog receiver front end, ADC, and microcontroller unit should be integrated in one chip as a system on chip (SoC) to improve the modules integration in the echo signal processing link of the UIS. In this way, miniaturization and low cost can be achieved, and high quality and low noise signal transmission ensured.
  - Reducing the use of components is an important way to improve the IC integration. Shared circuit modules and small size capacitors (e.g., MIM capacitors) may be used in the detail circuit design. Novel compact and high-performance circuit structures should be further developed. Digital calibrations for error optimization and linearity improvement should be used as far as possible, rather than analog.

2. Ultra-low-power ultrasonic ADCs should be developed.
  - Adopting of advanced fabrication processes means that ultra-low supply voltage can be used, which therefore reduces the power consumption of the ICs for UIS. This is also in good agreement with improving the integration of ultrasonic ICs. However, ultra-low voltage IC design technologies, such as current foldback, quasi-floating gate, etc., should be utilized.
  - Nanowatt-level power consumption UIS ADCs are expected in the future. Ultra-low power IC design techniques, including bulk-driven, sub-threshold conduction, and current-reuse techniques, etc., can be adopted in the circuit design.
  - Using as few devices or circuit modules as possible is another way to reduce the chip power consumption. Novel low-power circuit structures adopting circuit multiplexing scheme, inductor-less circuit structure, etc., should be further developed.

3. High resolution/speed ADCs should be investigated.
  - High resolution ADC can be used for the developed UIS, such as the color Doppler imaging. Novel Σ-∆ ADC with resolution of higher than 24-bits and tens of MHz bandwidth should be further developed.
  - Large bandwidth (high speed) ADC would be utilized to meet the requirement of high frequency UIS. Pipelined structure can be adopted, however, at the cost of more power consumption and chip area. A time interleaving scheme can be considered to greatly improve the data conversion rate.
  - Hybrid ADC that can meet the requirements of both resolution and speed at the same time should be further developed.
  - Digital correction technology needs to be developed to suppress or shape the mismatch noise generated by PVT variations, and therefore improve the linearity performance of the overall ADC. The dynamic element matching (DEM) technology is a very good choice and should be widely used in the circuit design.

## Figures and Tables

**Table 1:** Performance summary of the recently proposed ADCs for UIS.

Performance	Malki et al. [37]	Shu et al. [35]	Zhu et al. [41]	Yoon et al. [49]	Zhang et al. [48]	Li et al. [47]	Kaviani et al. [56]	Akter et al. [59]	Mao et al. [60]	Song et al. [67]	Zhang et al. [68]
Architecture	SAR	SAR	SAR	DI-DT * Δ-Σ	CT * Δ-Σ	MASH Δ-Σ	Pipelined	Pipelined-Split	Pipelined-Split	NS * Pipe-SAR	Pipe-SAR
Application	General UIS	—	General UIS	Biomedical Instrument- ation	Biomedical Ultrasound Beamformer	Color Doppler UIS	3D UIS	General UIS	General UIS	General UIS	—
Technology	40 nm LPCMOS *	55 nm CMOS	90 nm CMOS	65 nm CMOS	65 nm CMOS	35 nm CMOS	250 nm CMOS	40 nm CMOS	65 nm CMOS	28 nm CMOS	130 nm CMOS
Supply (V)	1.1	1.2	1.2	1.0	1	3.3	2.5	1	1.2	1	1.2/1.0/0.8
BW * (MHz)	—	0.001/0.004	—	0.01	15	8/20	10	25.6	50	40	
FS * (MHz)	40/80	1	100	1.28	1200	320	160	53	100	600	50/30/10
Area (mm2)	0.0656	0.072	0.18	0.22	0.16	24	4	0.76	0.38	0.016	0.22
Power Diss. *(mW)	1.45/2.86	0.0157	3	0.0248	6.96	544	330	9	32	2.56	1.32/0.56/0.12
SNDR (dB)	56.85/54.2	101/96.1	56.6	80.4	74.3	106/91.3	54.3	66	68.5	75.2	69.1/71.0/71.2
SFDR (dB)	69/65.12	105.1/105.1	71	—	—	—	—	77.3	84.4	87.1	80.7/80.0/81.5
ENOB (bits)	9.15/8.7	16.5/15.7	9.1	13.1	12.0	17.9/14.8	8.73	10.7	11.1	12.2	11.2/11.5/11.5
FoM * (fJ/step.)	63/85	0.17/0.29	54.7	2.21	1.42	6.95/59.6	4857	102	145	0.9	11.2/6.44/4.14

* BW: bandwidth; FS: sampling frequency; Power Diss.: power dissipation; FOM: figure of merit; FOM (fJ/step.) = Power/2ENOB*Fs; DI-DT: dual-integrating discrete time; CT: continuous-time; NS: noise shaping; ENOB: effective number of bits.
